# Phenomenological reduction and quantitative psychology: a conflict in the study of self-consciousness

**DOI:** 10.3389/fpsyg.2025.1650407

**Published:** 2026-01-05

**Authors:** Hu Ren

**Affiliations:** School of Humanities, Southeast University, Nanjing, China

**Keywords:** self-consciousness, quantitative psychology, phenomenological reduction, quantitative research, qualitative research

## Abstract

This paper explores the methodological tensions between quantitative psychology and phenomenological reduction in the study of self-consciousness. Quantitative psychology, historically modeled on the natural sciences, treats self-consciousness as a psychological attribute and operationalizes it through standardized scales. It emphasizes causal relationships between self-consciousness and other factors and primarily adopts a third-person perspective. Despite its contributions, this approach faces persistent challenges, including contextual dependence, constructed measurement tools, and the lack of natural measurement units. In contrast, phenomenology, originating with Husserl, emphasizes the intrinsic, pre-reflective dimension of self-consciousness present in all conscious experience. Unlike quantitative psychology, phenomenology contributes by clarifying meaning prior to causal explanation, recognizing the indispensability of the first-person perspective while acknowledging the value of third-person analysis. From the phenomenological standpoint, contextual dependence is not a limitation, and the aim is not to criticize the absence of natural measurement units in quantitative research, but to clarify the true meaning of the scores and the constructed units from the perspective of lived experience.

## 1 Introduction

Throughout the development of modern psychology, the quantitative method has occupied a central position, with its scientific legitimacy heavily reliant on the operation of measurement. From the late 18th century to the mid-20th century, thinkers in the fields of philosophy and the natural sciences began attempting to establish a mathematical foundation for psychology (Nuttgens, 2023). According to Johann Friedrich Herbart (1776–1841, as cited in (Leary, 1978, p. 118), psychology needed to adopt a mathematical form to achieve genuine scientific status. Although his approach was criticized for relying on arbitrary numerical assignments that lacked an objective basis (Leary, 1978), it still represented an important step toward mathematizing psychology.

Historically, psychology has largely emulated the methodology of the natural sciences (Englander and Morley, 2023), with psychologists especially keen on issues of replicability (Earp and Trafimow, 2015). After World War II, quantitative research gradually became the mainstream in psychology (Michell, 2002). However, entering the 21st century, quantitative psychology has increasingly encountered what has been termed a “crisis of confidence” (Botella and Durán, 2019; Earp and Trafimow, 2015; Oberauer and Lewandowsky, 2019). In recent decades, growing difficulties in quantitative research have led to a renewed interest in qualitative approaches (Marchel and Owens, 2007). Proponents of qualitative research acknowledge the undeniable contributions of quantitative methods in psychology, but argue that an exclusive commitment to quantification is fundamentally unscientific (Michell, 2011).

The development of social sciences led to growing recognition of the limitations of the positivistic view of science, fostering the rise of phenomenological, existential, hermeneutic, and postpositivist approaches, and prompting critiques of science’s claims to certainty, its exclusion of subjectivity and cultural context, and the narrowness of its methodological orientation (Wertz, 2015, p. 267). This paper discusses the conflict between phenomenology—particularly the method of phenomenological reduction—and quantitative psychology in the study of self-consciousness against this background.

With respect to phenomenology, Husserl (1990, pp. 18–19) regarded it as both a science and a method or attitude. Similarly, Heidegger (1962, p. 50) asserted that “The expression ‘phenomenology’ signifies primarily a methodological conception.” Phenomenology is the study of “pure” consciousness (Wertz, 2023b). It is often described as an investigation into how phenomena appear in our experience, how we perceive and understand them, and what they mean within our subjective consciousness (Neubauer et al., 2019). Phenomenology begins with an inquiry into the self or subjectivity (Zeiler et al., 2025). It can be defined as the scientific study of lived experience, turning attention toward experience itself and adopting a phenomenological attitude that shifts from a natural stance toward the world to examining how the world appears within consciousness (Lundh, 2020b).

The structure of this paper is organized as follows: section 2 introduces self-consciousness and the problems of measurement in quantitative psychology; section 3 elaborates on Phenomenological reduction as an alternative perspective on the study of self-consciousness; section 4 analyzes consciousness and self-consciousness from a phenomenological perspective; section 5 discusses the conflict between quantitative psychological research and phenomenological reduction in relation to self-consciousness, as well as the potential support for improving quantitative research with the help of phenomenological insights; section 6 provides a brief conclusion.

## 2 Self-consciousness and the problems of measurement in quantitative psychology

### 2.1 The definition, types, and scale-based operationalization of self-consciousness

In psychology, self-consciousness is often understood as the process through which individuals direct attention toward themselves (Duval and Wicklund, 1972). “Self-consciousness is defined as the act of focusing one’s cognitive reflection specifically on oneself” (Trudeau and Reich, 1995, p. 699). Self-consciousness reflects a dispositional tendency for individuals to direct attention toward various aspects of themselves (Panayiotou and Kokkinos, 2006).

Classic research distinguishes three forms of self-consciousness—private self-consciousness, public self-consciousness, and social anxiety (Cardeña et al., 2022; Fenigstein et al., 1975; Scheier and Carver, 1985). Private self-consciousness refers to the tendency to focus on the hidden, personal aspects of the self, such as one’s private beliefs, values, aspirations, and feelings, whereas public self-consciousness refers to the tendency to focus on aspects of the self that are visible to others and influence how one is perceived, such as behavior, mannerisms, and expressive qualities (Scheier and Carver, 1985). These traits exhibit meaningful psychological correlates: public self-consciousness is associated with social anxiety, while private self-consciousness is often linked to depressive tendencies (Panayiotou and Kokkinos, 2006). High levels of private self-consciousness may enhance resistance to misleading information, whereas high public self-consciousness heightens sensitivity to social scrutiny (Buss, 1980, pp. 47–61). Self-awareness and self-consciousness are related concepts but empirically distinct: self-awareness is a transient, situational state of self-focus, while self-consciousness is a stable, habitual trait of self-directed attention (Buss, 1980, pp. 19–20). Longitudinal studies indicate that self-consciousness shows year-to-year stability, supporting its classification as a personality trait (Davis and Franzoi, 1991). Self-consciousness is widely treated in quantitative research as a stable psychological attribute.

On this foundation, researchers have attempted to operationalize self-consciousness using standardized psychometric tools. Fenigstein et al. (1975) addressed longstanding ambiguity by distinguishing private and public self-consciousness and developing the Self-Consciousness Scale (SCS), transforming self-consciousness into a measurable variable. This work provided a foundation for subsequent research in social and personality psychology (Burnkrant and Page Jr, 1984; Carver and Scheier, 1978; Franzoi and Brewer, 1984; Heinemann, 1979).

Subsequently, Mittal and Balasubramanian (1987) contended that the Self-Consciousness Scale (SCS) encompasses a more complex structure than originally proposed, comprising distinct subdimensions of self-reflectiveness and internal state awareness within private self-consciousness, as well as multiple differentiated facets within public self-consciousness. Grant et al. (2002) further developed the Self-Reflection and Insight Scale (SRIS). By distinguishing between the two independent dimensions of self-reflection and insight, they provided a more refined operationalization of private self-consciousness, enhancing the scale’s reliability and predictive validity.

### 2.2 The problems of measurement in quantitative psychology

With the continuous refinement of measurement scales, psychologists have advanced quantitative investigations of self-consciousness across contexts, yielding substantial findings. Despite the operationalization of self-consciousness via standardized scales, fundamental questions remain about whether psychological attributes—self-consciousness being one example—can truly be quantified.

Although psychological attributes are commonly assumed to be measurable, this assumption has been persistently challenged throughout the history of psychology (Markus and Borsboom, 2011). Supporters of measurability argue that psychological attributes can be treated as “real objects” independent of the cognizer, much like the measurable entities in physics. They believe that it is possible to develop a universally applicable system for the standardized measurement of such attributes. Although psychologists acknowledge that psychology lacks a rigorous and universal system of measurement like that of physics, many still hold that certain attributes—such as anxiety, attitudes, and cognitive abilities—are quantifiable (Kyngdon, 2013). Opponents, however, argue that quantitative psychology is built on a theoretical assumption: namely, that psychological attributes are measurable. This issue has sparked considerable debate in the academic literature (Guyon et al., 2018).

In this regard, Michell contends that psychometricians have never seriously examined whether psychological attributes can actually be measured; instead, they have simply assumed so. Furthermore, he argues that the quantitative hypothesis has never been convincingly tested, and that the inherent immeasurability of psychological attributes has been deliberately concealed by flawed measurement concepts (Michell, 2000; Michell, 2004). As such, Michell famously labeled psychometrics, and quantitative psychology more broadly, a form of pathological science. According to him, unlike normal science, which corrects itself through critical thinking, psychometrics disguises the fact that psychological attributes are not measurable, thus rendering the falsity of the quantitative hypothesis difficult to detect and endowing the field with a pathological tendency (Michell, 2000).

Trendler (2009) argues that discussions about measurement must distinguish between extensive quantities (e.g., length, mass) and intensive quantities (e.g., temperature). The former are additive; the latter are not and can only be comparatively assessed. Psychological attributes, he argues, are more akin to temperature than mass, they can be compared as “more” or “less” but cannot be meaningfully added together. If psychological quantities do exist, they must be intensive rather than extensive (Kyngdon, 2013). From this view, psychological attributes must be measured indirectly via observable indicators. However, unlike physical phenomena, psychological phenomena are difficult to control or manipulate with the same precision. That is, psychological attributes are not inherently unmeasurable; rather, given current methodological and technological constraints, our ability to control or manipulate psychological phenomena does not yet match the precision achievable in the physical sciences.

Kyngdon (2013) adopts a more cautious stance, arguing that the core difficulty in psychological measurement lies not merely in technical limitations (as Trendler suggests), but in two fundamental issues: the lack of extant quantities and the absence of descriptive theories of psychological systems. The lack of extant quantities refers to the fact that psychological attributes, unlike physical quantities such as length, cannot be continuously and systematically varied or precisely manipulated in the same way. Take anxiety, for example: when we assign it a score from 0 to 100, we cannot be sure whether 60 points of anxiety truly represents twice the intensity of 30 points. In this sense, psychological “measurement” often amounts to numerical assignment rather than a genuine quantification of an underlying magnitude (Michell, 1997). The lack of descriptive theory means that for constructs like “anxiety” or “self-esteem,” psychology often relies on vague definitions and questionnaire scoring, without structural theories to explain how these states arise from stimuli or influence behavior, let alone build replicable and testable systems of measurement. In other words, the concepts associated with psychological attributes are highly abstract, and such abstraction is not grounded in a reliable foundation, resulting in definitional vagueness. As a result, although psychology claims to reject metaphysics, psychometrics remains metaphysically laden, with a persistent gap between what is actually observed and what is claimed to be measured—this gap being papered over by metaphysical assumptions (Michell, 2020).

Furthermore, Martin (2003) emphasizes that psychological phenomena are historically and socially constructed, and inherently imbued with moral and political significance. From this perspective, psychological attributes should be understood not as objective, mind-independent entities, but rather as emergent properties shaped by social context and the individual subject (Guyon et al., 2018). Given the contextual dependence of psychological attributes, what is more crucial than the question of whether they can be measured is that their true meanings must first be clarified before attempts at measurement.

In sum, the claim that psychological attributes are measurable faces at least three major challenges:

1. **Contextual dependence**: Psychological attributes are deeply shaped by language, context, social environments, and cultural traditions. The quantification of psychological traits often overlooks the social nature of measurement itself.
2. **Constructed measurement tools**: Measurement instruments and concepts are themselves human-made. Researchers may define constructs like “anxiety” or “self-esteem” differently, and these theoretical constructs are inherently constructed (Uher, 2021), leading to incompatibility across scales.
3. **Lack of natural measurement units**: Unlike physical attributes, psychological attributes do not have natural units or scales. There is no objective standard for “one unit of anxiety,” nor any empirical basis for saying that 60 units of anxiety is twice as much as 30.

Despite these three challenges, this paper maintains that the claim that psychological attributes can be measured is still reasonable to some extent. The crucial issue is not to deny the possibility of measuring psychological attributes altogether, but to recognize that quantification is appropriate insofar as the qualities that inevitably underlie all measurement are secured through proper methods. Phenomenological reduction may offer a suitable foundation for establishing such qualities.

## 3 Phenomenological reduction: an alternative perspective on the study of self-consciousness

As discussed in section 2, quantitative psychological research faces at least three major challenges when studying self-consciousness. Among these, the first challenge—contextual dependence—highlights the limitation of quantitative methods in capturing the inherently situated nature of psychological experience. Self-consciousness does not exist in isolation but emerges within social, cultural, and environmental contexts, which are often overlooked by standardized measurement instruments.

Phenomenology, particularly Husserl’s phenomenological reduction, offers an alternative perspective for addressing this challenge. According to Ashworth (2006, pp. 17–18), Husserl’s phenomenology was fundamentally aimed at furnishing a rigorous foundation for the various scholarly disciplines, an objective pursued through the elucidation of the meanings of their most basic concepts. In phenomenology, one of the key methods for clarifying fundamental concepts is phenomenological reduction.

In everyday life, we rarely pay attention to how the world is presented to us through consciousness. Instead, our interest typically lies in the already-given world and the entities within it (Gutland, 2018). This orientation toward the world is known as the natural attitude—a stance in which individuals engage with daily affairs within the familiar world they take for granted (LeVasseur, 2003). Within the natural attitude, people habitually accept the existence of an external world without question. In other words, when operating under the natural attitude, one presumes the existence of a real, objective world independent of oneself and acts accordingly in relation to others and the environment.

Phenomenological reduction begins with the suspension (epoché) of the natural attitude. It requires us to bracket all assumptions regarding the existence and nature of the external world; that is, to put them in parentheses. The true meaning of suspension is to exclude the natural, objective, and unreflective approach to the world (Hamill and Sinclair, 2010). This bracketing of the entire world, including ourselves as experiencing subjects, is a prerequisite for entering Husserl’s transcendental phenomenology (Gutland, 2018). Unlike scientific methods that suspend belief in isolated experiences, Husserl’s approach suspends all assumptions, including those about consciousness itself (LeVasseur, 2003). The aim is not to deny the existence of the external world but to suspend unexamined judgments and critically reflect upon them (Uljée, 2023). Through suspension, the object of inquiry is no longer the objective thing itself, but how it is given in consciousness, that is, how it appears in subjective experience. Phenomenological reduction enables researchers to focus entirely on the way the world is presented in immediate experience (Wertz, 2021, p. 132). Following the suspension of the natural attitude, eidetic reduction is employed to grasp the essential structures of phenomena. Eidetic reduction involves free imaginative variation—systematically varying a particular experience or object in imagination to discern the invariant elements that constitute its essence (Kim et al., 2020). Following Husserl, phenomenological reduction is broadly acknowledged throughout the phenomenological tradition.

## 4 Consciousness and self-consciousness from a phenomenological perspective

Phenomenology, originating with Husserl, offers a conception of self-consciousness that differs markedly from that of quantitative psychology. This section examines the phenomenological perspective by exploring the relationship between consciousness and self-consciousness.

While the phenomenological tradition encompasses a wide range of perspectives and should not be seen as entirely homogeneous, there is a notable convergence among its major figures concerning the relationship between consciousness and self-consciousness. Prominent phenomenologists—including Husserl, Scheler, Heidegger, Merleau-Ponty, Sartre, and others—have argued that the experiential dimension of consciousness is inherently characterized by a tacit or pre-reflective self-consciousness (Zahavi, 2006, p. 273). In other words, every conscious experience is implicitly “for oneself,” carrying an intrinsic first-personal quality, even before any reflective or deliberate awareness of oneself as the subject of that experience. This foundational insight highlights that self-consciousness is not merely a higher-order or reflective phenomenon, but an essential aspect of consciousness itself.

It has been noted that the idea of a self-referential component in conscious mental states has a long-standing philosophical tradition (Gennaro, 2006). This tradition traces back to Aristotle and was later emphasized by Brentano, who argued that every mental act inherently involves an awareness of itself (Brentano, 1995; Caston, 2002).

According to Husserl (1977, p. 159), “at any time a reflection directed toward the identical I is possible: and this I is the subject of all lived experiences and the subject for all its objects as a pole of unity of its intentionalities; but it is not itself a lived experience.” In Husserl’s view, the self (I-pole) is the structural subject unifying the stream of experience, while self-consciousness is the manner in which this self manifests within experience; thus, the self serves as a prior condition for self-consciousness rather than being identical with it. According to Heidegger (1982, p. 159), “The self is there for the Dasein itself without reflection and without inner perception, before all reflection.” In other words, according to Heidegger, self-consciousness in each person’s existence is a priori and primordial, arising directly with existence. It is a form of “prereflective self-awareness” that is part of existence itself, rather than something constructed through rational or psychological activity. According to Sartre (1956, p. 74), “the first condition of all reflection is a pre-reflective cogito. This cogito, to be sure, does not posit an object; it remains within consciousness. But it is nonetheless homologous with the reflective cogito since it appears as the first necessity for non-reflective consciousness to be seen by itself.” Sartre here points out that for consciousness to be able to reflect on itself, there must first exist a direct and natural self-awareness, that is, the pre-reflective cogito. This form of consciousness does not posit any object, yet it is the necessary condition for reflective consciousness to arise.

Zahavi emphasizes the distinction between pre-reflective self-awareness and reflective self-awareness in conscious experience. “To put it differently, self-consciousness has two different modes of existence, a prereflective and a reflective. The first has priority since it can obtain independently of the latter, whereas reflective self-consciousness always presupposes prereflective self-consciousness” (Zahavi, 2006, pp. 277–278). Pre-reflective self-awareness is an immediate, implicit form of self-experience that is non-objectifying (not taking oneself as an object) and non-conceptual (not mediated by concepts), whereas reflective self-awareness explicitly thematizes one’s own consciousness in a relational and conceptual manner (Zahavi, 2020, p. 35). “Thus, pervasive prereflective self-consciousness is definitely not identical with total self-comprehension, but can rather be likened to a precomprehension that allows for a subsequent reflection and thematization” (Zahavi, 2006, p. 278).

Phenomenology’s stance on the relationship between consciousness and self-consciousness appears to be very similar to that of higher-order theories of consciousness. But in fact, phenomenology and higher-order theories differ fundamentally in their accounts of the relationship between consciousness and self-consciousness (Zahavi, 2006, p. 276). Higher-order theories generally claim that a mental state becomes conscious only when it is represented by a further reflective or monitoring state, thereby treating consciousness as, at least in part, an extrinsic property of the mental state. Specifically, Carruthers (1998) argues that phenomenal consciousness cannot exist independently of self-awareness; rather, it constitutively involves higher-order thoughts about one’s own mental states. In contrast, phenomenology emphasizes that pre-reflective self-consciousness is an intrinsic feature of experience itself. “When one is prereflectively self-conscious one is not aware of oneself as an object that happens to be oneself, nor is one aware of oneself as one specific object rather than another. Rather, my prereflective access to myself in first-personal experience is immediate and nonobservational and nonobjectifying” (Zahavi, 2006, p. 280). From this perspective, every conscious state already carries a built-in dimension of self-manifestation, independent of any additional higher-order representation. This distinction highlights that, whereas higher-order theories rely on a form of meta-representation to account for consciousness, phenomenology locates self-consciousness within the structure of consciousness itself, as an immediate, non-reflective aspect of experience.

In sum, phenomenologists generally hold that self-consciousness is not an external or independent entity, but an intrinsic dimension present in every conscious experience.

## 5 The conflict between quantitative psychological research and phenomenological reduction

Phenomenological reduction conceives self-consciousness as an intrinsic dimension present in every conscious experience, whereas quantitative psychology treats it as a measurable entity. This divergent understanding underlies the methodological and epistemological tensions between the two approaches.

### 5.1 Causality and meaning

According to Giorgi (1992), there are two fundamental principles of human understanding: meaning and causality. Meaning and causality can be seen as pertaining to different domains: meaning generally relates to consciousness and intentionality, often requiring understanding, whereas causality typically concerns objects and their mechanical interactions, which require explanation (Cloonan, 1995, pp. 114–115).

The quantitative research path in psychology tends to treat self-consciousness as a psychological mechanism that can be defined, measured, modeled, and predicted, thus fitting within the empirical scientific framework of causal explanation. Since natural sciences have progressively established epistemological superiority since Newton and enjoy privileged academic status and resource allocation, psychology seeks to enhance its legitimacy and position within the academic community by borrowing natural science methods (Nuttgens, 2023), aiming to explain observed phenomena through causal explanatory theories (Uher, 2021). Since Fenigstein et al. (1975) distinguished three forms of self-consciousness—private self-consciousness, public self-consciousness, and social anxiety—and developed the Self-Consciousness Scale (SCS), psychology has measured individuals’ self-consciousness using scale-based methods and, on this basis, investigated the relationships between self-consciousness and other factors. For example, as noted in Section 2.1, Buss (1980, pp. 47–61) suggested that high levels of private self-consciousness may enhance resistance to misleading information, whereas high public self-consciousness increases sensitivity to social scrutiny.

From a phenomenological standpoint, self-consciousness comprises both prereflective and reflective dimensions. Quantitative psychology, however, primarily operationalizes reflective, conceptual self-consciousness through self-report instruments, leaving the prereflective dimension mostly overlooked. This methodological omission can be understood in relation to what phenomenologists describe as the “forgetting of the lifeworld.” For Husserl (1970, p. 50), the lifeworld refers to the world in which we live through bodily and intuitive experience—the concrete spatiotemporal environment of actual things, unmediated by the geometrical or mathematical idealities imposed by formal science. Husserl (1970, pp. 48–49) observes that already in Galileo’s time a crucial shift occurred: the mathematically constructed world of idealities came to be tacitly regarded as the only genuine reality, thereby displacing the perceptually given, experientially grounded lifeworld. Consequently, the original meaning of self-consciousness, which inherently includes a prereflective dimension, is progressively obscured in scientific approaches that prioritize formalized, abstract, and reflective measures. As Husserl (1970, p. 48) remarks, “Thus natural science undergoes a many-sided transformation and covering-over of its meaning.” Recognizing this gap opens the possibility of developing integrative measurement approaches that can more faithfully align empirical methods with the structural features of lived experience.

In addition, Husserl (1970, p. 31) argues that the universal causal structure of the intuitively experienced surrounding world allows for the formulation of hypotheses, the use of inductive reasoning, and the prediction of unknown aspects of its past, present, and future. In other words, Husserl does not reject universal causal outcomes. However, it is crucial to note that such causal structures are always understood within the lifeworld; they are empirical and experiential, and abstraction or mathematical idealization should be grounded in this experiential basis. Phenomenology emphasizes the significance of measurement and quantification themselves, and by revealing the true meaning of numerical relations, it reconstructs the meaning of numbers as constituted within experience (Wendt, 2024, pp. 91–92). However, before explaining causal relationships between self-consciousness and other factors through quantitative means, we should first clarify the original meaning of self-consciousness. As Van Manen (2016, p. 15) points out, “The meaning of human science notions such as ‘truth, method, understanding, objectivity, subjectivity, valid discourse,’ and the meaning of ‘description, analysis, interpretation, writing, text,’ etc., are always to be understood within a certain rational perspective.” The formation and expression of self-consciousness always involve a prereflective dimension; they are embedded in this shared world and realized through specific perspectives, and thus cannot be separated from mediating structures such as language, embodiment, and social interaction. Therefore, while quantitative methods can model causal relationships, such analyses must be grounded in a prior, phenomenologically informed understanding of the meaning of self-consciousness. In this sense, the analysis of meaning must take precedence over causal explanation.

In sum, quantitative psychology has overlooked the prereflective dimension of self-consciousness, whereas phenomenology contributes by revealing the importance of this prereflective dimension in the study of self-consciousness. This means that the phenomenological approach still holds great potential in psychological research and requires further exploration to establish a rigorous foundational discourse for psychology (Wendt, 2024, p. 79).

### 5.2 Third-person perspective and first-person perspective

In the study of self-consciousness, as shown in Section 2, quantitative psychology primarily measures self-consciousness through the Self-Consciousness Scale (SCS) and subsequent revised instruments, which represents a third-person perspective aiming to transcend subjectivity by constructing universally valid measurement systems. By representing the world externally and emphasizing replicability, generalizability, and standardization, quantitative psychology transforms subjective experience into a system of observable and controllable data (Wackers and Schille-Rognmo, 2022).

Phenomenological psychology, in contrast, emphasizes a first-person perspective, returning attention to subjectivity and the pre-reflective experience of consciousness. From this viewpoint, self-consciousness is not an object within experience but the foundational structure that makes experience itself possible (Gallagher, 1997; Neubauer et al., 2019). Husserl argued that the object cannot be separated from the experienced object-subject (Davidson, 2003), and that all access to the world occurs through consciousness, constituting a first-person perspective (Tuffour, 2017). Phenomenology thus prioritizes the description of lived experience, exploring the internal structures and meanings of consciousness, rather than attempting to quantify or externalize it.

Importantly, phenomenological psychology does not limit itself to the first-person perspective. Recent developments have highlighted the importance of the second-person perspective, in which the researcher engages empathically with participants to understand subjective experience within interpersonal interaction (Englander and Morley, 2023). This perspective is particularly valuable for capturing the subtleties of consciousness as it is expressed in social contexts, emphasizing intersubjectivity rather than an object-subject dichotomy. Methods such as micro-phenomenological interviews exemplify this approach, providing highly detailed accounts of experience while integrating the other’s perspective. Here, the researcher acts as a co-experiencer rather than a detached observer, bridging the gap between subjective experience and scientific inquiry.

In addition, phenomenology does not exclude the third-person perspective, as Husserl (1970, p. 262) argued that human beings, both individually and collectively, function as subjectivity for the world while simultaneously being situated within it in an objective and worldly manner. Merleau-Ponty (1962, p. x) likewise emphasizes that humans do not possess an isolated “inner self”; rather, they are fundamentally situated in the world, and self-knowledge arises only through their engagement with it.

In sum, from a research perspective, the contribution of phenomenology to the study of self-consciousness resides in its provision of a rigorous foundation for examining self-consciousness from a first-person perspective. Without the first-person perspective, we cannot access any objective reality; that is, the only way to approach objectivity is through subjectivity. Any objective knowledge of the world necessarily involves subjective access to that world. Without the involvement of first-person perspective, perception or knowledge construction would not be possible (Lundh, 2020a). In this sense, phenomenological reduction demonstrates that truth is variable and relative by introducing the first-person perspective (Sadala and Adorno, 2002).

### 5.3 Quantitative and qualitative research

Husserl (1977, p. 2) observed that “from the beginning, psychology was unable to resist the temptation of naturalism, of an extrinsic imitation of the model of natural science.” Since its inception as a scientific discipline, psychology has sought precision and systematic rigor through laboratory experimentation and quantitative measurement (Giorgi, 1985, p. 1). By modeling itself on the natural sciences, psychology has achieved a certain level of empirical success, yet this orientation has also led to deep methodological and philosophical tensions.

Giorgi (2006, p. 46) argues that although psychology has approached consciousness empirically through various theoretical assumptions, such efforts have inevitably distorted the very phenomenon under investigation. Similarly, Kockelmans (1987, p. 9) points out that since the eighteenth century, empirical psychology has been confused by the mistaken notion that its scientific method should emulate the physico-chemical sciences. According to Kockelmans (1987, p. 11), the “psychical” includes the ego and all experiences inherently connected to it—perceiving, thinking, willing—phenomena that cannot be meaningfully captured through methods designed for inanimate matter. Malcolm likewise argues that consciousness cannot be reduced to physical processes, emphasizing its subjectivity and irreducibility (Armstrong and Malcolm, 1984, pp. 45–46). Gutland (2023) argues that quantitative methods are indispensable in scientific research, but they often overlook the qualitative features of the experiential world.

These philosophical considerations reveal the limitations of the quantitative paradigm. As Wertz (2024) argues, psychological research cannot rely solely on experimentation, causal hypothesis testing, or mechanistic reductionist explanations. Although these methods serve the natural sciences well, they are insufficient for grasping the complexity, contextuality, and subjectivity inherent in psychological phenomena.

In response to this impasse, phenomenological psychology emerged as a distinct methodological and philosophical alternative. Giorgi—widely regarded as the most influential figure in phenomenological psychology in the Americas—developed a systematic descriptive method grounded in Husserl’s and Merleau-Ponty’s phenomenology. Giorgi (1970, pp. 171–172) argued that psychology, while traditionally modeled on the natural sciences, should instead be rooted in the life-world, the realm of lived human experience, rather than abstracted from it. According to Giorgi (1982, pp. 337–338), psychological knowledge possesses “empirical generality” rather than universal lawfulness; it cannot be evaluated using the objective criteria of the physical or rational sciences. His approach established psychology as a human science, with a research program that reflects the discipline’s existential and experiential dimensions (Cloonan, 1995, p. 109).

Parallel developments in the Dutch school of phenomenological psychology further reinforced this position. Kockelmans (1987, p. 18) maintained that phenomenological psychology provides the necessary foundation for conducting an exact empirical psychology—an aim long pursued but distorted by efforts to mimic the physical sciences. Ashworth (2006, pp. 18–19) similarly emphasized that phenomenological psychology is not about reporting empirical results but about establishing the fundamental principles of psychological inquiry, thus offering a secure basis for future research.

This shift also coincided with the formal emergence of qualitative research as a recognized methodological category in the 1980s (Rennie et al., 2002). Wertz (2019) argues that because measurement presupposes qualitative meaning—empirical quantities cannot be interpreted without qualitative grounding—qualitative analysis forms the foundation upon which quantitative methods depend. From this perspective, phenomenological psychology does not strip psychology of scientific legitimacy; rather, freed from naïve positivism, it becomes integrated within a universal transcendental philosophy (Wertz, 2023a). As Wertz (2023a) further notes, positive sciences provide only provisional knowledge, requiring ongoing reflection and revision; thus, psychology must remain open to continuous methodological renewal.

### 5.4 The difficulties of quantitative research and phenomenology’s contributions

As noted in Section 2, this paper points out that the study of self-consciousness from the perspective of quantitative psychology faces at least three major difficulties: contextual dependence, constructed measurement tools, and lack of measurement units. Building on these difficulties, the following discussion explores how phenomenology may offer insights into and advance our understanding of these issues.

First, from the standpoint of phenomenological reduction, contextual dependence is not considered a flaw. Phenomenology emphasizes that self-consciousness is inherently situated within the lifeworld, encompassing social, cultural, and environmental contexts. Phenomenology, as a qualitative research method (Neubauer et al., 2019; Sloan and Bowe, 2014), prioritizes the description of lived experience, exploring how consciousness unfolds through temporality, embodiment, space, and meaning. In qualitative research, the relationship between meaning and context is analogous to the relationship between the sample and the population in quantitative research (Englander, 2019). Contextual dependence provides the conditions for phenomenological reduction to explore the prereflective structure of self-consciousness and the original meaning of experience. By attending to pre-reflective experience, phenomenological approaches reveal the ways in which self-consciousness is inseparable from the contexts in which it emerges. Rather than attempting to isolate variables from context, phenomenology allows researchers to examine the dynamic interplay between subjective experience and its surrounding world. This perspective provides a nuanced understanding of contextual dependence, highlighting the relational and situational factors that quantitative measures often overlook.

Second, from the standpoint of phenomenological reduction, constructed measurement tools are unavoidable. The key is not to reject them for being human-made, but to examine the historical and conceptual processes through which they have been formed. As Husserl (1970, pp. 40–41) notes, the art of measuring is not merely a technical procedure but a continual practice of refining precision toward ever greater perfection. This means that measurement is not a purely objective technical activity but an ongoing practice that continually revises itself in the pursuit of greater precision; it inherently involves human thought, judgment, and construction, and requires a continual return to lived experience itself. Moreover, phenomenology—as a major branch of philosophy—is often described as a movement, a dynamic and evolving form of thought (Davidsen, 2013). Knowledge, from the phenomenological standpoint, is constituted through developmental processes (Sadala and Adorno, 2002). To this day, phenomenology remains an open-ended system of thought and a self-critical method that continually renews itself throughout its historical development, characteristically resisting the dogmatization of seemingly secure conclusions (Wertz, 2023b).

Third, from the phenomenological standpoint, the lack of natural measurement units may represent the most fundamental difficulty faced by quantitative psychology in the study of self-consciousness. However, this does not imply that self-consciousness cannot be quantified; rather, it indicates that the units used are not as clearly defined or intrinsically given as those for physical quantities such as length. For example, in self-consciousness scales, respondents answer a series of statements or questions, each assigned a conventional score reflecting a constructed rather than natural metric of self-consciousness, and the total score is used to quantify an individual’s self-consciousness. As a result, such measurements may have limited replicability across studies, contexts, or populations. This limitation does not, however, invalidate the legitimacy of quantitative approaches. Replicability cannot serve as a definitive boundary between science and non-science, as even in physics it is not always determinative (Earp and Trafimow, 2015). Moreover, the replication crisis is not unique to psychology; it also affects fields like biomedicine (Loscalzo, 2012). Therefore, from the phenomenological standpoint, the important task is not to criticize the lack of natural measurement units in quantitative psychology, but to clarify, as clearly as possible from the perspective of phenomenological reduction, the true meaning of the scores in self-consciousness scales. As Husserl (1970, p. 58) states, “Of first importance for this task, however, is the reflection on the original meaning of the new sciences.”

In summary, phenomenology can, while maintaining its philosophical foundation, be combined with quantitative analysis to provide a more comprehensive research perspective (Martiny et al., 2021). Phenomenology can deepen the metrological understanding of statistical procedures and help psychology to utilize them instead of being dependent on them (Wendt, 2024, p. 91). The challenges faced by quantitative psychology in studying self-consciousness suggest that a purely quantitative approach is insufficient for fully understanding this phenomenon. In recent years, the growth of qualitative research has signaled a shift in psychology toward broader and more inclusive methodologies (Nuttgens, 2023), highlighting the methodological compatibility of phenomenological reduction and quantitative psychology. Quantitative and qualitative research are not inherently antagonistic. Even in mathematics, quality and quantity are often intertwined and cannot be completely separated (Saint-Mont, 2012).

## 6 Conclusion

The study of self-consciousness demonstrates that quantitative and phenomenological approaches provide distinct yet complementary insights. Quantitative methods enable causal analysis and systematic investigation but often overlook the prereflective and contextually situated dimensions of self-consciousness. Phenomenology, in contrast, prioritizes first-person experience and the meaning of lived experience, revealing aspects of self-consciousness that remain hidden to purely quantitative investigation. The integration of phenomenology and quantitative psychology offers the potential to achieve a more comprehensive and nuanced understanding of self-consciousness, bridging subjective experience with empirical analysis. Greater synergy between phenomenological perspectives and quantitative techniques promises a pluralistic methodology capable of capturing both the experiential and measurable dimensions of self-consciousness, advancing the field toward a fuller, more rigorous understanding of the human mind.
